# Characterizing Topological Properties of Brain Functional Networks Using Multi-Threshold Derivative for End-Stage Renal Disease with Mild Cognitive Impairment

**DOI:** 10.3390/brainsci13081187

**Published:** 2023-08-10

**Authors:** Rupu Zhang, Xidong Fu, Chaofan Song, Haifeng Shi, Zhuqing Jiao

**Affiliations:** 1 School of Computer Science and Artificial Intelligence, Changzhou University, Changzhou 213164, China; 2 Department of Radiology, The Affiliated Changzhou No.2 People’s Hospital of Nanjing Medical University, Changzhou 213003, China

**Keywords:** end-stage renal disease, mild cognitive impairment, functional brain network, multi-threshold derivative, sparrow search algorithm optimized support vector machine

## Abstract

Patients with end-stage renal disease (ESRD) experience changes in both the structure and function of their brain networks. In the past, cognitive impairment was often classified based on connectivity features, which only reflected the characteristics of the binary brain network or weighted brain network. It exhibited limited interpretability and stability. This study aims to quantitatively characterize the topological properties of brain functional networks (BFNs) using multi-threshold derivative (MTD), and to establish a new classification framework for end-stage renal disease with mild cognitive impairment (ESRDaMCI). The dynamic BFNs (DBFNs) were constructed and binarized with multiple thresholds, and then their topological properties were extracted from each binary brain network. These properties were then quantified by calculating their derivative curves and expressing them as multi-threshold derivative (MTD) features. The classification results of MTD features were compared with several commonly used DBFN features, and the effectiveness of MTD features in the classification of ESRDaMCI was evaluated based on the classification performance test. The results indicated that the linear fusion of MTD features improved classification performance and outperformed individual MTD features. Its accuracy, sensitivity, and specificity were 85.98 ± 2.92%, 86.10 ± 4.11%, and 81.54 ± 4.27%, respectively. Finally, the feature weights of MTD were analyzed, and MTD-cc had the highest weight percentage of 28.32% in the fused features. The MTD features effectively supplemented traditional feature quantification by addressing the issue of indistinct classification differentiation. It improved the quantification of topological properties and provided more detailed features for diagnosing cognitive disorders.

## 1. Introduction

End-stage renal disease (ESRD) is a type of renal failure that requires long-term dialysis or kidney transplantation [1]. It is usually accompanied by multiple-organ dysfunction and central nervous system abnormalities in addition to renal failure [2]. This can cause memory impairment [3], cognitive control abnormalities [4], and emotional damage, along with other cognitive disorders [5]. Cognitive impairment is a common comorbidity in ESRD cases [6]. Uremia, thiamine deficiency, hypertension, hemodialysis (HD), transplant rejection, and electrolyte imbalance are some of the usual causes for the onset of cognitive impairment. For example, some research has shown that 30% to 60% of ESRD patients suffer cognitive impairment during HD treatment [7]. Mild cognitive impairment (MCI) is likely to later develop into dementia, which significantly affects the quality of life and health level of patients [8]. Certain cognitive training and rehabilitation treatments can effectively delay the onset of dementia for MCI patients. In some cases, patients can even recover to a state close to that of normal individuals [9]. Thus, investigating the brain-network topology of patients with ESRD with MCI (ESRDaMCI) aids in acquiring a more comprehensive understanding of how various pathological conditions impact the brain’s network. Furthermore, it facilitates the development of cognitive training and rehabilitation treatments, offering valuable guidance for clinical interventions [10].

Neuroimaging technology is a precise tool that is utilized for studying the structure, function, and connectivity of the brain in patients with brain diseases [11]. Previous studies have shown that voxel-based morphometry, surface-based morphometry, and diffusion tensor imaging have identified gray [12] and white matter defects [13] in ESRD patients. Furthermore, various analysis methods also can help to identify brain metabolism and function abnormalities in ESRD patients receiving HD treatment. These methods include arterial spin labeling [14], magnetic resonance spectroscopy [15], and single photon emission computed tomography [16]. Moreover, fractal analysis can be utilized for revealing the complexity and dynamics of EEG signals in ESRD patients to fully comprehend the function and connectivity of brain networks. These neuroimaging technologies are of significant value in identifying the pathological and physiological mechanisms of neurologic complications in ESRD patients and identifying potential imaging biomarkers, and they provide beneficial guidance for clinical treatment.

In recent years, research has utilized resting-state functional magnetic resonance imaging (Rs-fMRI) to explore the impact of neurological and psychological damage on brain function in ESRD patients. This revealed abnormalities in intrinsic brain activity that were observed and the disruption of both intra- and inter-regional connected networks [11]. A brain functional network (BFN) describes the interaction between brain function and structure, and it can describe the connectivity throughout the entire brain [17]. Rs-fMRI studies take the entire time series of the resting state as a basis for BFN analysis, which describes the connectivity level across the entire brain. Recent studies have shown that brain neural activity is time varying. The study of time-varying functional brain networks (TV-FBNs) can provide a more comprehensive understanding of the operational mode of the entire brain [18] and serve as a useful diagnostic tool for brain diseases.

Over the years, there have been many studies on the analysis of BFN characteristics by researchers. The method of applying graph theory to analyze brain networks has been widely used in brain imaging research. Bullmore and Sporns [19] introduced graph theory into the analysis of complex brain networks and described the graph theory parameters commonly used in brain functional networks (BFNs). These parameters can be used to explore the functional integration and functional segregation characteristics of brain networks from the perspective of brain connectivity. Jie et al. [20] considered the network topological structure information on different thresholds and improved the brain network’s ability to express topological structure information using a multi-kernel learning method.

In the past five years, Dai et al. [21] analyzed changes in the brain functional network of patients with depression through topological property analysis. They also examined the network cost function at different thresholds when analyzing network properties and obtained the optimal threshold for the evolution model of the brain network in patients with depression. Bian et al. [22] extracted brain network multidimensional persistent features based on persistent homology with multi-threshold filtering (MTF) and identified brain connectivity patterns specific to Alzheimer’s disease. Xi et al. [23] constructed dynamic hypergraphs and introduced hypergraph popular regularization and L1-norm regularization terms into the brain network construction model, extracting hypergraph features for the classification of patients with ESRDaMCI and health control (HC). Zhang et al. [24] used graph theory to extract the AUC value of the topological properties within the sparse threshold range as a feature using the GRETNA toolbox to predict the degree of cognitive impairment in ESRD patients and HC individuals.

The above studies depended directly on the graph theory parameters of brain networks for topological properties within an absolute threshold range, reflecting the relationship between numerical values and static features. If the changes in brain networks filtered through multi-threshold and the details of these properties are ignored, the model’s ability to recognize features will be limited to individual numerical values or total values, and global detailed analyses of brain networks will be affected, thus impacting classification performance. This study characterizes the relevant topological properties reflecting the integration and differentiation of brain networks and establishes a new classification framework for ESRDaMCI to address these issues. The DBFNs are constructed from preprocessed functional magnetic resonance imaging (fMRI) data. Each window of the weighted DBFN undergoes binarization using a set of linearly increasing thresholds. Then, the multi-threshold derivative (MTD) features are extracted from the resulting binary brain network. The sparrow algorithm (SSA) is introduced to optimize the parameters in the support vector machine (SVM) kernel function and classify them according to four parameters that evaluate their classification performance. The established framework is performed to identify specific details of topological properties related to the integration and differentiation of brain networks of MTF, and to more accurately recognize ESRDaMCI patients in classification.

Inspired by Bian‘s research [22], this study focuses on graph filtering for brain-network analysis. It employs the novel approach of utilizing the topological attribute derivative curve with multiple thresholds to assess differences between two groups of brain networks and incorporate them as classification features. Bian et al. achieved positive results by observing changes in the number of connected branches in brain networks through threshold filtering and extracting their derivative curve features for classification. The four extracted topological attributes effectively reflect the integration and differentiation characteristics of the brain. Therefore, a detailed analysis of these attributes, using multiple thresholds and extracting derivative curve features, may enhance the distinction of differences in brain functional networks and elevate the accuracy of classification between ESRDaMCI and HC groups.

## 2. Materials and Methods

### 2.1. Date Acquisition and Processing

The fMRI data from 51 cases of ESRDaMCI were collected from the Affiliated Changzhou No. 2 People’s Hospital of Nanjing Medical University. Their educational level ranged from 5 to 19 years, with a mean education of 11.25 ± 3.15 years. They met the following inclusion criteria: (1) no other category of neuro-psychiatric disorders; (2) no history of cardiovascular or cerebrovascular diseases; (3) no use of antipsychotic medications. Furthermore, a total of 39 individuals was recruited into the HC group. The educational level of the HC group ranged from 5 to 19 years, with a mean education of 9.73 ± 3.85 years. They met the following inclusion criteria: (1) no personal or family history of neuro-psychiatric disorders; (2) no history of head trauma or loss of consciousness; (3) no alcohol or drug abuse; (4) no claustrophobia; (5) no internal implants. All participants in the experimental study were right-handed and had a history of good physical health with no significant statistical differences in terms of age, gender, and education level. Five and three subjects with excessive head movement were excluded from each of the two groups. This study was conducted with the approval and supervision of the Ethics Committee of the Affiliated Changzhou No. 2 People’s Hospital of Nanjing Medical University (approval number KY039-01). All participants provided written informed consent before the testing.

In terms of clinical assessment of cognitive function, the Montreal Cognitive Assessment (MoCA) scale was used, which is a commonly used tool in this field. The MoCA scale contains 11 items for detecting various cognitive domains, such as language, memory, abstract thinking, visual–spatial skills, executive function, attention and concentration, calculation, and orientation [25]. In these cases, compared with other cognitive function assessment scales, the MoCA has better completion rates and better sensitivity in identifying MCI in memory clinics [26]. The cognitive function of these ESRD patients was evaluated using the MoCA scale. The full score of the MoCA scale is 30 points, and a score of 26 or higher is considered normal, 18–26 mild, 10–17 moderate, and less than 10 severe. All neuropsychological tests were evaluated by a neurologist with 20 years of experience before MRI scans. The mean score of ESRD patients diagnosed with MCI was 21.30 ± 2.75 points, and the specific demographic information is shown in Table 1 [10].

A GE Discovery MR 750W 3.0T superconducting MR scanner with 32-channel head–neck combined coil was used for acquiring the Rs-fMRI images of the subjects. Prior to scanning, all subjects underwent routine MR examination, and image diagnostic physicians excluded cranial organic damage. The acquisition parameters included repetition time (TR) of 2000 ms, echo time (TE) of 40 ms, field of view (FOV) of 24 cm, flip angle (FA) of 90°, matrix size of 64 × 64, and slice thickness of 6 mm.

After collecting Rs-fMRI data, the raw data were preprocessed using the DPARSF toolbox [27]. Prior to preprocessing, the SPM8 toolbox (http://www.fil.ion.ucl.ac.uk/spm/ accessed on 11 October 2022) and DPARSF toolbox (http://rfmri.org/dparsf accessed on 13 October 2022) had to be installed. The following specific steps were performed: (a) Image format conversion: the DICOM format was converted to the NIFTI format by the DICOM Import function in SPM8; (b) Slice timing correction: it took some time for both the instruments and the subjects to reach a stable state, so the first ten time-points for each subject were removed before the remaining time series were used for subsequent processing; (c) Head motion correction and spatial normalization: a rigid registration method was applied to enhance consistency across all time points. The fMRI images were transformed into Montreal Neurological Institute (MNI) space. The normalized images were further processed by setting the Bound Box to [−90, −126, −72; 90, 90, 108] and Voxel Size to [333] to ensure consistency between images and reduce noise interference. (d) Spatial smoothing: full-width-at-half-maximum was applied to smooth the Gaussian kernel. (e) Linear drift removal. (f) Band-pass filtering: the frequency range was 0.01–0.08 Hz. (g) Brain segmentation: after removing covariates, each subject’s brain was divided into 90 brain regions by the Automated Anatomical Labeling (AAL) standard partition template [28], and the time series of each brain region was extracted for subsequent analysis.

### 2.2. Research Framework

**Figure 1:**
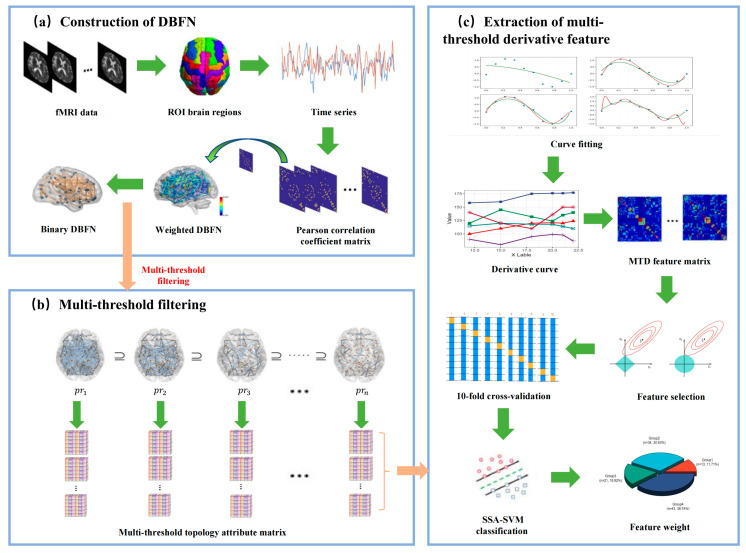
Research framework diagram.

Figure 1 shows the research framework. It mainly includes the following steps: (a) preprocessing of the original resting-state fMRI sample data to extract the time series of all ROI brain regions; (b) constructing the DBFN from the time series; (c) setting a linearly increasing threshold *pr* within the absolute threshold range; (d) thresholding the weighted brain network in each window to obtain the corresponding binary network by each *pr*; (e) extracting the topological property matrix (clustering coefficient, global efficiency, local efficiency, characteristic path length) of the binary network; (f) vectorizing the topological property matrix extracted from each binary network under each threshold in each window, fitting it to a multi-threshold receiver operating characteristic (ROC) curve, and calculating its derivative curve characteristics within the sparse threshold interval; (g) partitioning the training set and the testing set, taking the derivative curve within the absolute threshold range as the feature, and using lasso for feature selection; (h) partitioning the training set and the testing set by 10-fold cross-validation, training the SSA-SVM classifier on the training set, and then classifying and evaluating the classification performance on the testing set.

### 2.3. Construction of Dynamic Brain Function Network

There are many methods of constructing DBFNs, such as Pearson correlation coefficient (PCC), partial correlation, and Bayesian network. Through comparison, it has been found that constructing brain networks by PCC is more sensitive to the integration and segregation features of the brain and has better classification performance through comparison.

The BFN was constructed by applying pairwise PCCs, with different regions of the brain seen as nodes and PCC values as edges between them.

(1) $$p_{ij}=corr\{x(i),x(j)\}$$

where *x*(*i*) and *x*(*j*) respectively represent the time series of the *i*-th and *j*-th brain regions, and *corr* is the pairwise correlation coefficient between *x*(*i*) and *x*(*j*).

The DBFN divides the entire Rs-fMRI time series into multiple subsequences by sliding windows [29]. Specifically, assuming that the total number of time points in Rs-fMRI is *M*, it is divided into D=[(M−L)/s]+1 sub-sequences. Here, *L* is the length of the sliding window, *s* is the step size, and *P* is the number of brain regions. For the *d*-th window, the time series of the *i*-th brain region is defined as $x_{i}^{d}\in R^{L},\left(d=1,\ldots ,D\right)$, which is concatenated with the time series of other brain regions to obtain the time series matrix for the *d*-th window.

(2) $$X^{\left(d\right)}=\left[x_{1}^{d},x_{2}^{d},\ldots ,x_{p}^{d}\right]\in R^{L\times P}$$

A DBFN is constructed by calculating the PCC between time series within each time window. After centralizing and standardizing $x_{i}^{d}$, the correlation matrix of the *d*-th window BFN can be represented as $Z^{\left(d\right)}\approx {\left(X^{\left(d\right)}\right)}^{T}X^{\left(d\right)}$ and transformed into an optimization form:

(3) $$\underset{Z^{\left(d\right)}}{min}{\left\|Z^{\left(d\right)}-X^{{\left(d\right)}^{T}}X^{\left(d\right)}\right\|}_{F}^{2}$$

The correlation coefficient *r* approaches −1 or 1, resulting in smaller variance when taking PCC as functional connectivity. This can affect the analysis efficiency. The Fisher R–*Z* transformation to the correlation coefficients is conducted to ensure the stability of variance [4]. This transformation converts the correlation coefficients to the *z*-scale, producing coefficients that approach a normal distribution:

(4) $$FZ\left(r_{ij}\left(s\right)\right)≜\frac{1}{2}\ln\left(\frac{1+r_{ij}\left(s\right)}{1-r_{ij}\left(s\right)}\right)$$

where $r_{ij}\left(s\right)$ represents the PCC between regions of interest *i* and *j* at point *s*, and FZ(⋅) represents the Fisher R–*Z* transformation.

### 2.4. MTD Feature Extraction and Feature Selection

It is a straightforward and feasible approach to construct a BFN using the Pearson correlation. However, it presents a particular issue, namely that all ROIs in the brain are fully connected [30,31]. The reason is that the Pearson correlation is sensitive to both direct and indirect connections and may be interfered with by noise signals. A threshold strategy is introduced to convert a weighted BFN into a binary network by selecting a threshold to address this issue. This approach allows us to analyze the connection structure rather than the connection strength. Nevertheless, this also has a limitation in that there is often uncertainty in threshold selection for diagnosis of different clinical populations, which makes difficulties in network comparison and feature extraction.

The integration and differentiation of brain networks refer to two basic modes of information processing in the brain. Integration refers to integrating different sources of information, experience, and knowledge into a coherent whole, forming an inductive cognitive understanding of things. Differentiation refers to breaking down a whole into its component parts in order to better understand their details and differences, forming a deductive cognitive understanding of things [32]. There are various topological properties in graph theory indices, such as clustering coefficient, betweenness centrality, global efficiency, local efficiency, and small-world properties [19]. Four important topological properties related to the integration and differentiation of brain networks are selected: clustering coefficient (cc), global efficiency (Eglobal), local efficiency (Elocal), and characteristic path length (Lp).

Once the binary brain networks are extracted for each window of each participant, the topological property parameters for each binary brain network within each window are extracted using the Brain Connectivity Toolbox (BCT). The topological attribute values corresponding to each discrete threshold point are computed within the absolute threshold range for each topological attribute. Subsequently, functional data analysis (FDA) is applied to fit these discrete points in a ROC curve [33]. Then, FDA extracts the feature of the fitting curve by combining B-spline basis functions and a roughness penalty factor [34], thereby reflecting the network metric information contained in MTD from a curve perspective. This can be mathematically defined as shown in Formula (5).

(5) $$y_{mt}=\sum_{k=1}^{j}c_{k}\phi_{k}\left(t\right)+\varepsilon_{j}=c^{T}\phi \left(t\right)+\varepsilon_{j}$$

where ϕ represents the B-spline basis function, *c* is the penalty coefficient, and the residuals $\varepsilon_{j}$ are statistically independent.

However, the fitting curve obtained using this method only interpolates the datapoints and may not be accurate enough. When computing the fitting curve, Formula (6) enables the extraction of significant curve features and reduces computational burden, thereby addressing the problem at hand. Additionally, it can find the optimal balance point in these two factors, making the resulting fitting curve more accurate and reliable.

(6) $$\min F\left(c\right)=\sum {\left[y_{j}-c^{T}\phi_{j}\right]}^{2}+\lambda \int {\left(c^{T}\varphi_{j}\right)}^{2}dt$$

where ${\left[y_{j}-c^{T}\phi_{j}\right]}^{2}$ represents the squared errors (SEs) of the residuals, and $\lambda \int {\left(c^{T}\varphi_{j}\right)}^{2}dt$ represents the roughness of the balanced curve.

As λ increases gradually, the balance curve becomes smoother and tends to be a straight line, and SE gradually decreases. Then, the changes and derivative characteristics of fitted curves can be tracked by employing the FDA method. The derivative curve characteristics allow more precise features to be extracted from a mathematical perspective, which can then be used to calculate differences in brain networks. For statistical analysis, the differences in features are verified between the ESRDaMCI group and the HC group using an independent-samples *t*-test. The lasso feature selection method has high generalization ability and avoids overfitting by adding a regularization term $L_{1}$. Then SVM is trained according to the calculated features.

### 2.5. Model Construction

With good generalization performance, SVM offers several advantages, including simplicity, ease of result comparison, and wide applicability in small-sample classification problems [35,36].

There are two very important parameters in the SVM model, namely, *C* and *Gamma*. *C* is the penalty coefficient, which determines the tolerance to errors. If the value of *C* is too high or too low, it can decrease the model’s ability to generalize. *Gamma* is a parameter in the RBF kernel function, which determines the distribution of data on the feature space after mapping. The size of *Gamma* affects the number of support vectors, and the number of support vectors affects the training and prediction speed of the model.

The classification performance of SVM is greatly influenced by the selection of its own parameters. In many studies, animal swarm algorithms are introduced into the classifiers for optimization [37,38]. The optimized SVM with SSA was adopted to enhance classification performance by optimizing the *C* and *Gamma* parameters of the SVM.

The SSA algorithm, inspired by biological behavior, is a swarm intelligence optimization algorithm based on the foraging and predator avoidance behaviors of sparrows [39]. For a population of sparrows,

(7) $$X=\left[\begin{array}{cccc} x_{1}^{1} & x_{1}^{2} & \ldots & x_{1}^{d}\\ x_{2}^{1} & x_{2}^{2} & \ldots & x_{2}^{d}\\ \ldots & \ldots & \ldots & \ldots \\ x_{n}^{1} & x_{n}^{2} & \ldots & x_{n}^{d} \end{array}\right]$$

where *n* represents the number of sparrow populations, and *d* represents the dimensionality associated with each sparrow individual.

The discoverers in the algorithm exhibit strong search ability and prioritize finding food, so their fitness values are also higher. They have a better global orientation ability than the explorers and can provide foraging directions for the population. Hence, there is a need to have a wider search range to search for food. Their position update is expressed as follows:

(8) $$X_{id}^{t+1}=\left\{ \begin{array}{lll} X_{id}^{t}\cdot \exp\left[\frac{-i}{\alpha \cdot T}\right], & if & R_{2}<ST\;\\ X_{id}^{t}+\Gamma \cdot Z, & if & R_{2}\geq ST\; \end{array}\right.$$

where $X_{id}^{t}$ represents the position of the *i*-th sparrow in the *d*-th dimension in the *t*-th generation of the population. *T* is the maximum number of iterations. α is a random number in the range (0, 1], and Γ is a random number following a normal distribution. *Z* represents the size of i×d. $R_{2}\in \left[0,1\right]$ and ST∈[0.5,1] represent the danger warning and safe values perceived by the sparrow, respectively.

Formula (8) shows that when $R_{2}$ is greater than the warning threshold *ST*, it indicates that some sparrows have already detected danger. Accordingly, the discoverers should move to a safe location; that is, they randomly move near the current position according to a normal distribution. An $R_{2}$ value less than the warning value *ST* indicates that the environment is safe, and the discoverer may expand their search actions accordingly. With an increase in the number of population generations, the range of values that $\exp\left[\frac{-i}{\alpha \cdot T}\right]$ can take will also decrease, and the distribution of values will become more uniform; that is, the value of each dimension of sparrow individuals will decrease accordingly.

Apart from the discoverer, the remaining sparrows in the population are all followers. The update of the followers’ positions can be described as follows:

(9) $$X_{id}^{t+1}=\left\{ \begin{array}{ll} \Gamma \cdot \exp\left[\frac{Xw_{d}^{t}-X_{id}^{t}}{}\right], & if\;i>\frac{n}{2}\\ Xp_{d}^{t+1}+\left|X_{id}^{t}-Xp_{d}^{t+1}\right|A^{+}\cdot Z, & otherwise \end{array}\right.$$

where $Xw_{d}^{t}$ represents the sparrow’s worst position in the *d*-dimensional space during the *t*-th iteration, while $Xp_{d}^{t+1}$ represents its optimal position during the *t +* 1-th iteration. The row vector ***A*** has either 1 or −1 for each of its elements, and $A^{+}=A^{T}{\left(AA^{T}\right)}^{-1}$.

This indicates that the pursuer is in a very hungry state when *i* is greater than *n*/2, at which point it will use random numbers drawn from the standard normal distribution and apply the exponential function with natural logarithms to increase its energy levels and meet its energy demands. If *i* is less than or equal to *n*/2, it will search a location adjacent to the current optimal position randomly. Within this process, the values of each dimension tend to remain peaceful since they only undergo minor changes that are relatively small in comparison to the current optimal solution.

Among the sparrow population, a certain percentage of sparrows, ranging from 10% to 20%, exhibits an alert mechanism. These sparrows have randomized, updatable positions that are renewed with every subsequent iteration. The formula for updating these specific positions is as follows:

(10) $$X_{id}^{t+1}=\left\{ \begin{array}{lll} Xb_{d}^{t}+\beta \left(X_{id}^{t}-Xb_{d}^{t}\right), & if & f_{i}\neq f_{g}\\ X_{id}^{t}+K\left[\frac{X_{id}^{t}-Xw_{d}^{t}}{\left|f_{i}-f_{w}\right|+\delta }\right], & if & f_{i}=f_{g} \end{array}\right.$$

where $Xb_{d}^{t}$ represents the current global optimal position, whereas β denotes the step control parameter. The direction of the sparrow’s movement is signified by the random number *K*, which is selected from the interval [−1, 1]. δ is a very small constant value to prevent division by zero. The mutation factor $f_{i}$ determines the fitness value of the *i*-th sparrow, while $f_{g}$ and $f_{w}$ indicate the overall optimal and worst fitness values, respectively, within the current sparrow population. In the event that a sparrow is in the alert state and inhabits the current global optimal position, it will relocate to a nearby location. Nevertheless, if the sparrow is not in the optimal position, it will flee towards the vicinity of the current optimal position.

Classification performance is evaluated by four metrics: classification accuracy (ACC), area under the receiver operating characteristic curve (AUC), sensitivity (SEN), and specificity (SPE) [40]. Classification accuracy (ACC) is defined as the ratio of the number of correct predicted labels to the total number of samples. AUC measures the probability that the classifier ranks a random positive sample higher than a random negative sample. Sensitivity (SEN) and specificity (SPE) represent the true positive rate and false positive rate. These evaluation metrics are calculated by true positive (TP), false positive (FP), true negative (TN), and false negative (FN).

## 3. Results

Considering the limited number of data samples, 10-fold cross-validation was employed to evaluate the performance of the proposed method [41]. Simply put, the sample data were divided into 10 parts, and experiments were conducted by taking 9 parts as the training data and 1 part as the testing data in turn. The training set cannot participate in the model testing process to avoid information leakage, which may lead to an improvement in model performance. The relationship and feature embedding between brain regions can be unified by constructing the DBFN.

### 3.1. Parameters Selection

The above method had multiple parameters, which made it impossible to directly find the optimal parameter combination through the grid search method. This study progressively determined the optimal parameter values for the classification model by a 10-fold cross-validation method. The best classification model was sought by the ESRDaMCI patient training set, and then the performance of the model was evaluated by observing its performance on the test set. This yielded the number of classifiers and test results corresponding to the sample size. Next, the average of the test results was calculated to evaluate the model’s performance. The model was repeatedly trained to determine the optimal hyperparameters to prevent data leakage. Ultimately, the model was tested by original samples under these determined hyperparameters.

The sliding window length (*L*) and step size (*s*) of DBFNs are important parameters that greatly affect the reliability of DBFNs. On that basis, the DBFNs of all participants were classified according to different values of *L* and *s* to determine the optimal values. In classification, ESRDaMCI patient subjects were considered to be positive samples, and HC subjects were considered to be negative samples.

In the experiment, 10 step sizes were set with *s* = 1, 2, …, 10, and 10 sliding window lengths were set with *L* = 10, 20, …, 100 [42]. Appropriate window lengths and step sizes can improve the classification performance and give more remarkable results. The optimal *L* and *s* values were determined by analyzing the classification performance of multiple parameter settings. Table 2 shows the best performing set of indicators and standard deviations in each window length, with the best classification performance highlighted in bold black.

The results showed that the classification performance was best when *L* was set to 85 and *s* to 3, which is consistent with the research conclusion of Xi et al. [23]. As *L* and *s* increase, the classification performance first improves and then deteriorates. It is necessary to choose the appropriate window length and step size, which is consistent with the conclusion of Li et al.’s [40] research. The possible reason is that a smaller window length cannot maintain the integrity of the information well, while a larger window length and step size might limit the dynamic information between brain regions over time.

### 3.2. Selection of Threshold Range and Threshold Step Size

In the classification process, the selection of the absolute threshold range has a significant impact on the classification results. In the comparative experiment, the ranges of 0.01 to 0.2, 0.01 to 0.35, 0.01 to 0.5, 0.01 to 0.65, and 0.01 to 0.8 were selected as the ranges for the absolute threshold, with a step size of 0.01. Table 3 shows that when the range of the absolute threshold was from 0.01 to 0.35, the MTD feature yielded the best classification performance, which is consistent with the research conclusion of Zhang et al. [43].

### 3.3. Analysis of MTD Features

**Figure 2:**
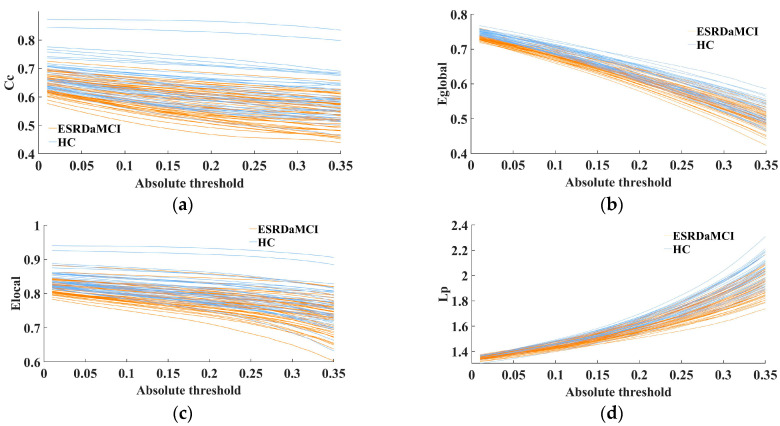
Fit curves of topological properties of two groups of subjects in MTF: (**a**) clustering coefficient; (**b**) global efficiency; (**c**) local efficiency; (**d**) characteristic path length.

The MTD features were quantified by the FDA method (as introduced in Section 2.5). The fitting curves of four topological attributes were then obtained for each subject by MTF, eliminating the need for threshold selection via graph theory. MTD enabled the extraction of brain network feature sequences in various threshold levels, which can retain and explore hidden network features of different thresholds, improving the consistency and interpretability of the analysis results. As shown in Figure 2, the analysis of multi-threshold ROC fitting curves for four topological properties revealed that the HC group consistently exhibited higher values compared to the ESRDaMCI group. Moreover, the trend of variation within the range of absolute thresholds (0.01–0.35) was more pronounced and divergent.

**Figure 3:**
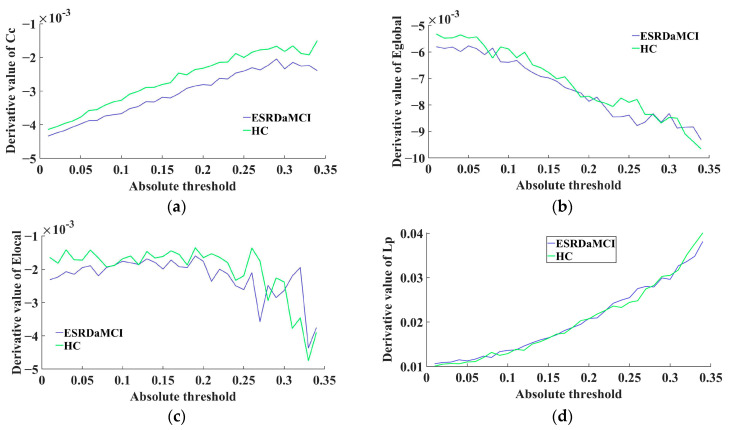
Comparison of MTD curves between two groups: (**a**) clustering coefficient; (**b**) global efficiency; (**c**) local efficiency; (**d**) characteristic path length.

The MTD curve of each subject was constructed after calculating the derivative values of the fitting curves. The average MTD curve of the two groups was calculated, as shown in Figure 3. The figure shows the average derivative curves of different topological attributes for the ESRDaMCI group and the HC group, where it was observed that the average derivative curve of the HC group tended to be higher most of the time. The above observations indicate that the brain network of HC was more sensitive to the change of threshold in the MTF, while ESRDaMCI patients were somewhat slower to respond to changes in threshold and needed more time to connect to the entire brain. From a statistical perspective, a significance test was performed on the MTD linear fusion features. The results revealed a significant difference between the ESRDaMCI group and the HC group (*p* = 0.02 < 0.05).

### 3.4. Classification Performance

**Figure 4:**
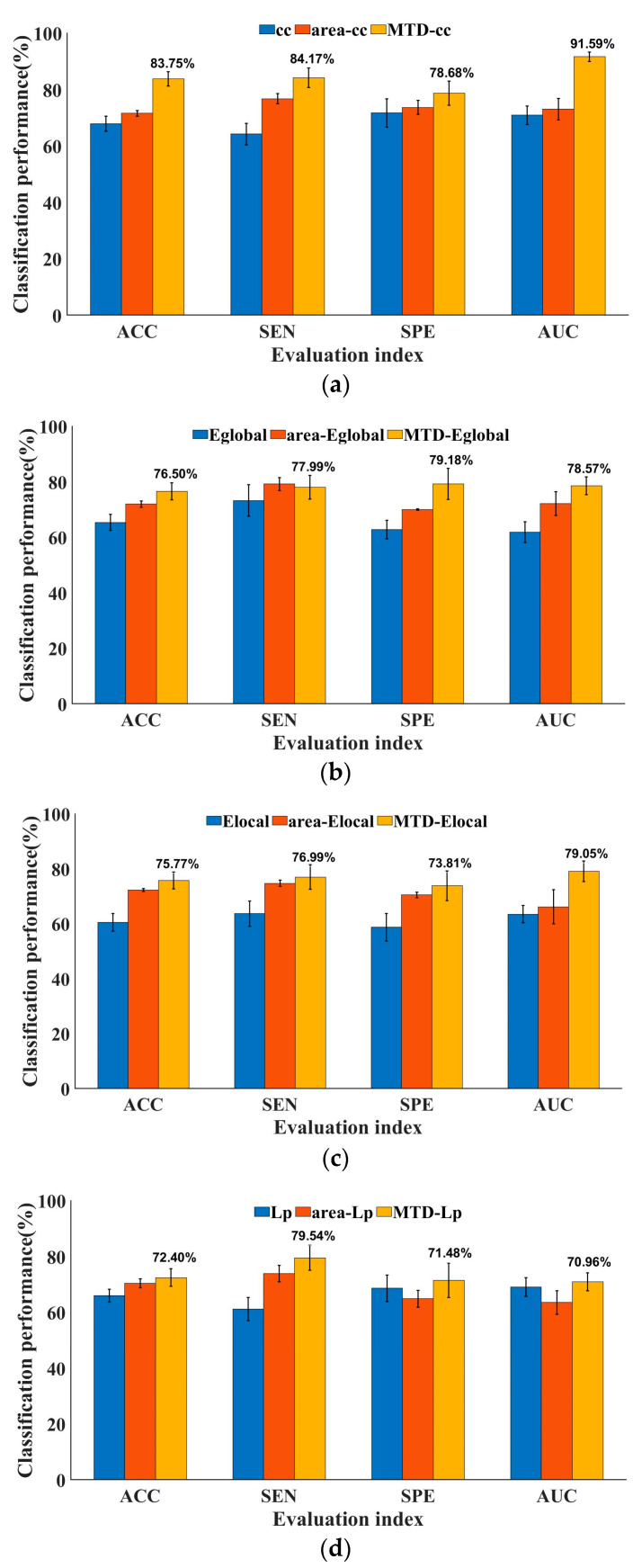
Performance comparison of ESRDaMCI classification based on various MTD features: (**a**) clustering coefficient; (**b**) global efficiency; (**c**) local efficiency; (**d**) characteristic path length.

As shown in Figure 4, four complex network measures, each correlated with the integration and differentiation of brain networks, were individually characterized by derivative curves of multi-threshold. The classifiers were evaluated by comparing them with the multi-threshold area under the ROC curves proposed in graph theory algorithms (namely area-cc, area-Eglobal, area-Elocal, and area-Lp) [24]. Moreover, traditional topological attributes [44] were also considered. The evaluation indicators, namely, ACC, SEN, SPE, and AUC, assessed the classification performance. From the figure, it can be seen that the classification performance of the four MTD features was generally better than the area under the multi-threshold curve features and traditional DBFN topological attributes in these four indicators. The classification accuracies were as follows: MTD-cc, 83.75 ± 2.53%; MTD-Eglobal, 76.50 ± 3.05%; MTD-Elocal, 75.77 ± 3.06%; and MTD-Lp, 72.40 ± 3.14%. They were all higher than their corresponding area features and traditional topological attributes, proving that mining the topological attributes of brain networks of MTF can effectively improve the differentiation between the ESRDaMCI group and the HC group.

The MTD-Fused feature was obtained by linearly fusing the MTD-cc, MTD-Eglobal, MTD-Elocal, and MTD-Lp features. It was compared with MTD-cc, MTD-Eglobal, MTD-Elocal, MTD-Lp, and edge connectivity features in terms of classification performance, as shown in Table 4. The classification accuracy of the traditional edge connectivity feature was 60.60 ± 3.33%. The fused feature was higher in classification performance in all four indicators than the unfused features, with a classification accuracy of 85.98 ± 2.92%, which was higher than the classification accuracy of the highest-performing single MTD feature, MTD-cc, with an accuracy of 83.75 ± 2.53%. This indicates that fusing the MTD features of the four important topological attributes can better reflect the integration and differentiation of brain networks for the ESRDaMCI group and the HC group, effectively improving the classification accuracy of the features. It clearly proves that the MTD features after linear fusion have better performance in classification.

### 3.5. Feature Weight

**Figure 5:**
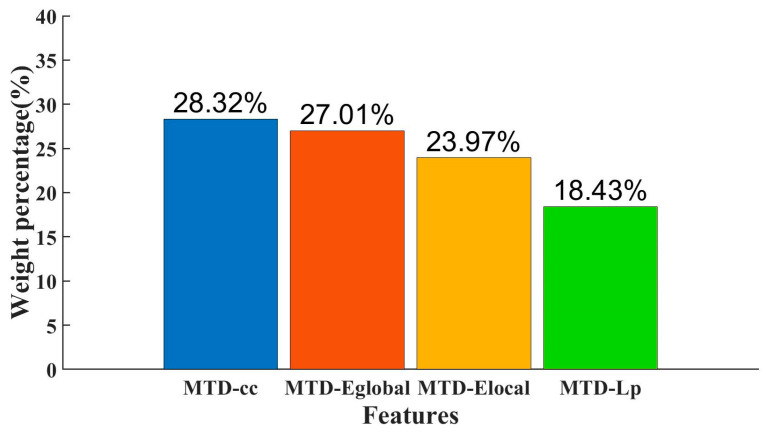
Percentage of feature weights.

Figure 5 shows the weights of individual MTD features in the classification and the percentage of the combined MTD feature weight. Among them, the MTD-cc feature has the highest percentage of the combined feature, accounting for 28.32%, corresponding to its classification accuracy of the individual MTD feature. Cc is an index of local network characteristics, reflecting the degree of interconnection between nodes in the brain network, paying more attention to the interconnection between nodes, and it can provide a detailed description of local characteristics of the brain network. Eglobal, Elocal, and Lp can reflect the information transmission efficiency and global characteristics of the brain network; they are indexes describing the overall network. In MTD, the local nodes in brain regions are more sensitive than the global nodes.

## 4. Discussion

In recent years, more and more researchers have conducted in-depth studies on diseases associated with mild cognitive impairment in epidemiology, clinical characteristics, neuroimaging [45], biological markers, disease mechanisms, neuropathology, clinical trials, and other areas, showing a strong interest. These challenges may be turned into opportunities for further exploration of the human brain with the development of new neuroimaging technologies. So far, there have been relatively few studies on ESRDaMCI classification, and most of the studies are based on the properties of the data, ignoring more detailed features. This study quantifies the relevant topological properties reflecting the integration and differentiation of brain networks based on MTD. Compared with general classification features, the ROC curve with multi-threshold and its derivative curve provide more comprehensive feature information for DBFNs that was provided by previous DBFNs. They reflect the dynamic characteristics of brain networks of multiple thresholds for ESRDaMCI patients and HC subjects.

Currently, there is related research analyzing multidimensional brain network connectivity characteristics and graph theory indices. For example, Rolls et al. [46] constructed a dynamic time-varying network based on time characteristics and used time-varying network-analysis methods to explore the abnormal dynamic time-varying characteristics of BFNs in the course of schizophrenia and ADHD. Parente et al. [47] analyzed the graph theory indicators of resting DBFNs with multi-threshold and the modular connections of brain networks. However, Rolls et al. [46] analyzed the construction of time-varying networks and intergroup differences in multi-dimensional brain networks descriptions. However, observing the BFNs solely were not persuasive to the dynamic functional connections of the time-varying process. Parente et al. [47] characterized static brain networks but did not consider the of time series dimension, which was not comprehensive enough for characterizing the network. Therefore, it is important to use multidimensional features to describe the properties of complex brain networks effectively.

It is worth mentioning that this study was inspired by Bian et al. [22]. In their study, the number of connected components in the brain network was extracted based on the graph filtering of persistent homology and characterized by derivative curves. Nevertheless, the variation in the number of connected components in brain networks of MTF is only a relatively rigid feature, which ignores many changes in the local network and lacks an overall consideration of brain integration and differentiation, which is somewhat one-sided. Moreover, a set of binary brain networks was obtained by applying MTD to DBFNs, and four relevant topological properties were extracted based on the integration and differentiation of binary networks. The change trend of these properties in MTF was fitted and analyzed, and then it was characterized by the derivative curve and finally used for classification. The extracted features can dynamically reflect the global and local characteristics of the brain, while the features extracted from Bian et al.’s study cannot capture the detailed local features of the brain network with multi-threshold.

However, the aforementioned studies still have some limitations. First, the experiment only used single-modality imaging (Rs-fMRI) and only considered BFNs, while the brain structural network also had many hidden features and details. Secondly, this study involved a large number of parameters, and the current strategy for parameter selection involved conducting manual comparative experiments. Finally, for the classification of the ESRDaMCI group and the HC group, only binary classification was considered, and there was a lack of transitional phases in the ESRDaMCI group and the HC group, leading to overly absolute classification results. To overcome these limitations, future studies will investigate a brain structural network and integrate it with a BFN for multi-modal fusion. Additionally, further studies on multi-modal brain networks will be conducted. New optimization algorithms will be introduced to facilitate optimal parameter selection for different datasets. Additionally, a multi-classification or scoring system will be employed for conducting a differential evaluation of brain networks, enabling a more detailed classification of the severity of cognitive impairments.

## 5. Conclusions

This study characterized the topological attributes of brain network as MTD features and established a new classification framework for ESRDaMCI. It expanded the utilization of inherent topological properties as classification features and addressed the constraints associated with threshold selection dependence. Four essential topological properties were selected, which reflected the integration and differentiation of brain networks, and their detailed features were extracted based on multi-threshold derivative curves. This significantly improved the accuracy of classification between the group of patients with ESRDaMCI and the HC group. The results demonstrated that MTD features exhibited higher accuracy after comparing various traditional topological properties and ROC curves with multi-threshold topological properties. After that, the MTD features were further linearly fused and compared with individual MTD features and connectivity edge features, reflecting that the fused features achieved the best performance in classification.

## Figures and Tables

**Table 1:** Demographic information.

Items	ESRDaMCI Group (*n* = 51)	HC Group (*n* = 39)	*t* $/\chi^{2}$	*p*-Value
Age **(** $\bar{x}\pm s$ **)**	50.05 ± 7.86	48.37 ± 6.59	1.079	0.251
Sex (male/female)	24/27	24/15	0.341	0.536
Education **(**$\bar{x}\pm s$ **)**	11.25 ± 3.15	9.73 ± 3.85	0.973	0.771
MoCA score **(**$\bar{x}\pm s$**)**	21.30 ± 2.75	27.27 ± 1.24	−13.728	0.000

ESRDaMCI: ESRD patients with mild cognitive impairment; HC: healthy subjects; a difference between two groups is considered statistically significant when the *p*-value computed from a statistical test is less than 0.05.

**Table 2:** Classification performance with different window widths and step sizes.

Parameters	ACC (%)	SEN (%)	SPE (%)	AUC
*L* = 10, *s* = 1	69.8145 ± 2.9756	71.2636 ± 4.3215	57.1225 ± 5.0852	0.6796 ± 0.0249
*L* = 20, *s* = 1	71.5749 ± 2.6566	70.3181 ± 3.9787	72.9815 ± 4.6674	0.7773 ± 0.0237
*L* = 30, *s* = 1	72.9857 ± 2.2747	74.5969 ± 3.3198	64.8148 ± 4.1679	0.7567 ± 0.0172
*L* = 40, *s* = 1	74.7921 ± 2.5568	77.1198 ± 4.1764	65.3048 ± 3.4141	0.7815 ± 0.0176
*L* = 50, *s* = 3	78.8728 ± 3.4125	80.0218 ± 4.1765	70.0854 ± 6.0286	0.8221 ± 0.0313
*L* = 60, *s* = 2	79.9497 ± 2.0519	80.0065 ± 2.4059	74.5783 ± 4.3582	0.8557 ± 0.0203
*L* = 70, *s* = 5	83.6330 ± 1.8352	83.3115 ± 2.4281	79.1737 ± 4.0828	0.8845 ± 0.0146
*L* = 80, *s* = 3	**85.9828 ± 2.9149**	**86.1002 ± 4.1113**	**81.5384 ± 4.2663**	**0.9351 ± 0.0161**
*L* = 90, *s* = 2	77.5066 ± 1.8927	79.6666 ± 2.7604	74.8689 ± 4.6902	0.8077 ± 0.0200
*L* = 100, *s* = 8	73.0201 ± 2.3719	71.8301 ± 2.9657	70.9527 ± 4.1789	0.7670 ± 0.0203

**Table 3:** Classification performance with different ranges for the absolute threshold.

Parameters	ACC (%)	SEN (%)	SPE (%)	AUC
0.01–0.2	74.0740 ± 2.3162	78.4313 ± 3.1987	56.4102 ± 4.0162	0.6907 ± 0.0138
0.01–0.35	**85.9828 ± 2.9149**	**86.1002 ± 4.1113**	**81.5384 ± 4.2663**	**0.9351 ± 0.0161**
0.01–0.5	78.8648 ± 2.7561	76.5882 ± 3.6924	79.4615 ± 3.1972	0.8261 ± 0.0182
0.01–0.65	70.8899 ± 2.3752	72.5490 ± 4.9238	66.1538 ± 4.8692	0.6626 ± 0.0152
0.01–0.8	66.0714 ± 2.2366	62.5490 ± 3.1365	70.4615 ± 4.2534	0.5184 ± 0.0212

**Table 4:** Performance comparison of ESRDaMCI classification using fused features.

Features	ACC (%)	SEN (%)	SPE (%)	AUC
Connections	60.6029 ± 3.3321	64.2048 ± 4.4825	57.8063 ± 5.4026	0.5913 ± 0.0311
Mtd-Lp	72.3982 ± 3.1392	79.5425 ± 4.5174	71.4758 ± 6.1055	0.7096 ± 0.0318
Mtd-Elocal	75.7744 ± 3.0562	76.9935 ± 4.4751	73.8120 ± 5.4223	0.7905 ± 0.0375
Mtd-Eglobal	76.5015 ± 3.0517	77.9956 ± 4.2267	79.1823 ± 5.6347	0.7857 ± 0.0321
Mtd-cc	83.7527 ± 2.5289	84.1656 ± 3.4824	78.6838 ± 4.2664	0.9159 ± 0.0172
Mtd-Fused	**85.9828 ± 2.9150**	**87.1678 ± 4.1113**	**81.5385 ± 4.2664**	**0.9352 ± 0.0161**

## Data Availability

The data presented in this study are available on request from the corresponding author. The data are not publicly available due to privacy and ethical reasons.
